# Searching for synergy from a combination of heterogeneous business models: measurement and assessment from the Polish software industry

**DOI:** 10.1016/j.heliyon.2019.e01970

**Published:** 2019-07-08

**Authors:** Jolanta Wartini-Twardowska, Zbigniew Twardowski

**Affiliations:** a University of Economics in Katowice, 1 Maja 50, 40-287, Katowice, Poland; b Silesian Higher School of Economics, Harcerzy Września 1939 3, 40-659, Katowice, Poland

**Keywords:** Business, Synergy potential, Business model combination, Value creation, Company group, Software industry

## Abstract

The authors propose a universal methodology for measuring and assessing the synergy potential when combining companies into a group, using the business model perspective. Based on interviews with company representatives, as well as consultations with Polish software industry experts, we have found 40 key variables which are characteristic for business models of resellers and integrators. The combination of business model concepts, of Balanced Scorecard and multi-criteria decision analysis, as proposed in the paper, allows the opportunities and threats (for strategic objectives to be reached within company group) in terms of business model variables, to be indicated more precisely. The results of the multi-criteria analysis of the decision making process show the reseller - integrator combination to create a high potential for market synergies and an average potential for improving operational processes. Present methodology generates the transparent information on how a business model works and how it stimulates the combination of companies. In particular, we provide the picture of synergy potential of the company group.

## 1 Introduction

Mergers and acquisitions (M&As), as a means of overcoming barriers to business development, have long been the subject of research and attracted the attention of the industry (cf., Haspeslagh and Jemison, 1991; Mitchell and Mirvis, 2001; Lynch, 2006; Zollo and Meier, 2008; KPMG, 2011). Although synergy effects have become one of the most important determinants of M&As, they have not been fully recognized (see Sirower, 1997; Capron, 1999; Mitchell and Mirvis, 2001; Mukherjee et al., 2004; Krishnan et al., 2009; Fiorentino and Garzella, 2015). Despite the crucial importance of synergies to M&As, the market is highly skeptical about the valuation of its estimated effect (Goold and Campbell, 1998). “Too high” acquisition premiums and mistakes in the integrating value chains of combined companies (Lee et al., 2006) or different organizational cultures (Hitt et al., 2001) contribute to the loss of synergy effects. On the other hand the market may see synergies in the announced combination owing to the complementarity of their resources, which, according to Larsson and Finkelstein (1999), may be an underappreciated source of value creation in M&As. Each of these arguments underlines that outlining the image of true synergies and their sources cannot be overestimated.

The existing literature on the techniques of evaluation or measurement of synergy in M&As presents narrow or wider proposals for their depiction. Narrow proposals are largely focused on: (a) synergy sources (Lubatkin, 1987; Kaplan, 2010); (b) factors to success (Cullinan et al., 2004; KPMG, 2011); (c) anomalies concerning large-company combinations (Sirower, 1997); (d) synergy management pitfalls (Fiorentino and Garzella, 2015); (e) M&A principles (Christensen et al., 2011); (f) ways of balancing the synergistic benefits (Markides, 2008); (g) systematic risk or reduction of risks (Chatterjee and Lubatkin, 1990; Chatterjee, 2007); (h) resource interactions (Chatterjee and Brueller, 2015); and (i) challenges in managing acquisitions (Haspeslagh and Jemison, 1991). The wider proposals for techniques to assess or measure synergies include: (a) models for measuring synergies in Pre-M&As (Ansoff, 1965; Wartini-Twardowska, 2014; Fiorentino and Garzella, 2015); (b) factors of failure (Haspeslagh and Jemison, 1991); (c) methods of valuation (Damodaran, 2006; Lenz, 2011); (d) model of conditions determining the success of M&As (Chadam and Pastuszak, 2013); and (f) classification of measures or identification of measures in the context of M&A performance (Zollo and Meier, 2008; Meglio and Risberg, 2010). On the one hand, there is a general consensus on methods for measuring synergy effects in the area of financial planning of investment projects. However, the focus on the financial perspective does not solve the problems of failures in M&As if the estimated values of financial ratios are based on questionable assumptions. One may partially concede that "if there are no true synergies between the merging firms in the first place, then even to high quality, low-cost implementation of the merger may lead to only negligible benefits" (Zollo and Meier, 2008, p. 60). However, decision-making in such circumstances is highly risky, even if the parties to the transaction are only concerned with the negligible benefits. On the other hand, the state of research on measuring synergies in the area of strategic planning is still inadequate. Garzella and Fiorentino (2014, p. 1196) claim that in "Theoretical and empirical research still lacks a common understanding of the effectiveness of synergy measurement in M&A". Their proposal of a simple model for measuring synergy effects increases the effects’ understanding by considering four factors: *type of synergy*; *size of synergy*; *likelihood of achievement*; and *timing of synergy*.

Nevertheless, these techniques have limitations: They do not allow us to understand how heterogeneous business models and their variables stimulate the synergy effects of the company group in the M&A process, and how, through potential opportunities to achieve strategic objectives of combining companies, the synergy effect of the group is achieved.

The basic objective of the paper is to present the original methodology of measurement and assessment of the synergy potential of the company groups operating according to the heterogeneous business models. Such an objective of the work has been described by the following partial objectives: (a) proposal for standard lists variables of IT business models, to be used in other sectors after their prior verification; (b) identification and systematization of variables within the components of business models; (c) attempt to determine the degree of convergence between the key variables of a business model and the strategic objectives in order to recognize the opportunities and threats for a company group.

Based on the contribution of the authors quoted in the article, we present an original proposal for a synergy measurement methodology that is a confident response to the tasks of measuring synergy in the light of strategic risk management in pre-M&As. We assume a priori that the synergy will result primarily from the creation of a unique business architecture and through continuous improvement of the operational/capital structure. Our methodology allows us to address all the factors of Garzella and Fiorentino (2014) model, including the last one (i.e., timing of synergies), by developing multiple merger scenarios. In addition, a more analytical insight into synergies is provided, as its foundation is based on the concept of the business model and the Balanced Scorecard. The main result of this research fills addresses an identified theoretical gap through a multi-stage methodology for measuring and explaining the synergy potential of a company groups that operating according to heterogeneous business models (TG1). As noted by Teece (2010), the business model concept has theoretical shortcomings with respect to economic and business fundamentals, and the state of recognition of most of the areas of the concept examined by Wirtz et al. (2016) is weak. Nevertheless, we have successfully addressed the following two cognitive gaps:

• Full identification and categorization of variable business models in the context of value creation (CG1);
• Recognizing potential strategic risks (in the form of opportunities and threats) and supporting value-oriented decisions for a combination of companies operating according to heterogeneous business models (CG2).

In addition, our research addresses the following practical gap (PrG): Combining the concepts of the business model and Balanced Scorecard to sketch a more accurate picture of the synergy potential of combining companies into a group (PrG1).

Regarding the first gap (CG1), we refer to Shin and Park's (2008, p. 334) argumentation that "The business model concept is ... vague and general" as well as Wirtz et al. (2016, p. 48), who stated that "business model research is still at an early stage” (Wirtz et al., 2016). In addition, the research on the forms and components of the business model has received scant attention (Wirtz et al., 2016). Owing to our clear view of the use of structural analysis of business models, we address CG1 by identifying and classifying the variables that make up the components of the business models.

The starting point for the second cognitive gap (CG2) is the conviction that the common treatment of the business model concept and value creation is insufficiently recognized (Jabłoński, 2013). The study of business models is an important topic for strategic management research because such models are sources of value and affect companies' possibilities for value creation and value capture (Amit & Zott, 2001, p. 2). Current articles even discuss "the idea behind a 'model' of value creation" (Arend, 2013) as an “alternative business model understanding" (Wirtz et al., 2016, p. 45). For instance, the use of SWOT (*Strengths*, *Weaknesses*, *Opportunities*, and *Threats*) analysis together with the business model canvas enables assessment and evaluation of the business model as a whole along with its components in the context of value creation. Osterwalder and Pigneur (2011) even elaborate on the advantages of such an analysis.

The combination of the business model and Balanced Scorecard concepts as well as the multi-criteria decision analysis proposed in the paper allows for a more accurate indication of opportunities and threats (of implementation of strategic objectives in a company group) in relation to the key variables of its business models. This proposal for measurement and assessment may be helpful in finding alternative solutions, mitigating threats, and finding innovations in the group's business models that will create real value. Only a few authors have so far used multi-criteria analysis when investigating the value system from the perspective of the business model, as noted by Wirtz et al. (2016). Surprisingly, the analyses of the synergy effect by assessing opportunities and threats as well as by using the business model concept and Balanced Scorecard have been wanting.

Addressing the practical research gap (PrG) is relatively easy because works that thoroughly investigate synergy have been largely non-existent, thus indicating the worth of the simultaneous use of the business model and Balanced Scorecard approach. We follow Kaplan's proposal, which, while presenting the Balanced Scorecard values, refers to sources of synergy according to four perspectives (financial; customer; internal process; and learning and growth) (Kaplan, 2010). Moving from structural analysis to analysis based on multi-criteria decision-making, we generate information about what is happening inside the business model (among its variables) and how business models motivate combination. Considering value creation, such information can be directly used in scenarios concerning future evolutionary directions of business models implemented by company groups. Therefore, this study allows us to propose a methodology that provides the industry (i.e., managers and auditors) with transparent information about business models. It facilitates us to discover a situation despite the potential conflict between business model variables and the strategic objectives of the group. This methodology is therefore a new and valuable way of understanding synergy.

The remaining paper is organized as follows. We start by presenting a literature review of studies covering information technology (IT) business models and their typologies as well as the contribution to synergy in M&A. Then, we describe the research methodology and data collection process. We include the parameterization of the business models from the software industry (Integrator and of Reseller with Added Value) arising from the identification of their lists of variables and structural analysis. This is followed by the findings on synergy, including the examined synergy potentials of a combination in form of company group consisting of heterogeneous business models and the "synergy road-map." The paper finishes with the conclusion and applications of the research.

## 2 Background

This section describes the state of knowledge based on the selected literature on business models, their classifications, and the synergy theories. From this literature review, a clear picture of the motivation of our research is drawn, particularly: (1) fields of study of business models; (2) similarities and differences in published frameworks in terms of classifications of business models in the software industry (Tables 1, 2, and 3); (3) defined concepts, and (4) gaps in the research discovered, which helped us formulate the methodology in section 3 to assess the potential synergies for value-oriented combinations of business models.

**Table 1:** Selected studies on frameworks classifying business models in the software industry.

Author	Types of research design and method/theory	Context/key research questions	Findings
Rajala et al. (2003)	Theoretical basis of the presented concept: company and business strategies. Exploratory qualitative study carried out using the case study methodology comprising interviews and observations to collect primary data. The research design framework was described as a multiple case study.	(1) What are the generic elements of business models in software businesses? (2) The object is to develop a framework for analyzing the business models of software companies.	Framework based on four dimensions: (1) Product strategy: from customer-oriented to standard offer (customized product/solution, product platform, uniform core product, modular product family, standardized on-line service); (2) Revenue logic (effort-, cost-, or value-based pricing, license sales and royalties, revenue sharing, hybrid models and loss-leader pricing, other, e.g., media models); (3) Distribution model: from centralized/collaborative to decentralized/transactional (direct contact with customers, reseller or agent model, republisher/OEM model, distributor or dealer model, partner network); (4) Services and implementation strategy: complex/tailored vs. standardized (IT consulting & customer-specific system work; system integration projects; software deployment; on-line services, self-service).
Rajala and Westerlund (2007)	Theoretical basis of the presented concept: **(1)** Transaction cost theory; **(2)** The resource-based view of the company; **(3)** Theories of industrial networks and interaction	(1) What kinds of assets and capabilities are essential to different business models in the software industry? (2) Which of them are developed internally and which are obtained externally?	Classification framework based on two dimensions: (1) Degree of involvement in customer relationships (from low to high); (2) Level of homogeneity of offering for multiple customers (from low to high). Four generic types of models: (1) Models oriented to close cooperation with the customer, offer of tailor-made solutions (high, low); (2) Models using a standard IT platform for building customer solutions (high, high); (3) Suppliers of specialized components, models labeled as “resource provisioning” (low, low); (4) Suppliers of “standard offerings,” or businesses that seek large numbers of customers and economies of scale (low, high).
Popp (2011)	**(1)** A semiformal approach to classifying and modeling business models by type, along with examples from three successful software companies; **(2)** Research based on the business model classification system developed by Weill (based on a study of approximately 1000 companies).	(1) What are the business models of successful software companies? (2) Can a business model leverage successful software to create a competitive advantage?	Classification framework based on three dimensions: (1) Type of goods or services (financial goods: cash and other assets; physical goods: physical products—durable and nondurable; intangible goods: software and intellectual property; human services: people's time and effort); (2) The business model archetypes (creator; distributor; lessor; broker); (3) The revenue model (one or more revenue streams). Fourteen generic types of models: (1) financial products: 1.1 entrepreneur; 1.2 financial trader; 1.3 financial lessor; 1.4 financial broker; (2) physical products: 2.1 manufacturer; 2.2 wholesaler or retailer; 2.3 physical lessor; 2.4 physical broker; (3) intangible products: 3.1 inventor; 3.2 IP distributor; 3.3 IP lessor; 3.4 IP broker; (4) specialized IT services: 4.1 n/a, 4.2 n/a, 4.3 contractor; 4.4. HR broker.
Schief (2014)	Value chain analysis: **(1)** Literature review in the area of software value chain concepts; **(2)** Delphi study to analyze the economic characteristics of the initial set of activities by experts' domain; **(3)** Clustering algorithms to build a value chain hierarchy. Empirical examples by applying the value chain to software companies, as a proof of concept. Empirical analyses based on the primary data, collected from a large-scale survey of software companies in Germany	**(1)** What are the primary activities of a software value chain*?;* **(2)** What are the characteristics (i.e., components and choice options) of a software business model?; **(3)** What are the software business model characteristics in today's software industry?	Framework based on five dimensions (1) Strategy (value proposition; investment horizon; value chain; degree of vertical integration, number of cooperation partners); (2) Revenue–financials (sales volume; revenue source; pricing assessment; payment flow structure; revenue distribution model); (3) Upstream–value configuration (software stack layer; platform; license model; degree of standardization; key cost driver); (4) Downstream–customers (localization; target customer; target industry; target user; channel); (5) Usage–value configuration (implementation effort; operating model; maintenance model; support model; replacement strategy).
Wartini–Twardowska, (2014)	Theoretical basis of the presented concept: (1) Company and business strategies; (2) Value chain management (Porter); (3) The concept of the structure of the business model by Osterwalder and Pigneur. Empirical analysis based on the primary data, collected from direct, unstructured, interviews with IT managers.	**(1)** What criteria for identifying business models will allow sufficient characterization of the mechanisms of creating added value in the software industry? **(2)** How can the structure and relations of business models be described defined based on selected identification criteria?; **(3)** Is it possible to characterize the business architecture of the chosen organization based on the generic business models of the software industry in terms of potential opportunities and threats to the development of its business within the group?	Multilevel classification framework: **LEVEL 1**: Two classification dimensions: (1) a five-element value chain of software industry (design; production; distribution, implementation; maintenance); (2) basic strategies for creating value-added (close integration with the customer; economies of scale; innovations). Five generic business models were obtained for each value-added strategy: Design (1.1 Independent IT advisor; 1.2 Auditor; 1.3 Business consultant); Production (2.1 Independent software vendor; 2.2 Software development company; 2.3 IT Lab); Distribution (3.1 Value-added reseller; 3.2 Distributor; 3.3 Challenger); Implementation (4.1 Integrator; 4.2 Freelancer; 4.3 Software house); Maintenance (5.1 Help Desk; 5.2 IT on demand; 5.3 Service provider). **LEVEL 2**: Classification of generic business models due to the structure of the value of characteristic variables and interactions for key components: market segments; value proposition; relationships; distribution channels; revenue streams; key resources, key processes; partners; cost structure. For each generic business model, a set of key attributes has been defined (i.e. variables that take values from a particular range) best characterizing its properties.

**Table 2:** INT and VAR business models in light of different classification frameworks in the software industry.

Authors	Dimensions of classification	Generic Model: INT	Generic Model: VAR
Rajala et al. (2003)	Product strategy	Customized product/solution	Product platform
	Revenue logic	Effort-, cost-, or value-based pricing	Revenue sharing, license resale
	Distribution model	Direct contact with customers	Direct contact with customers
	Service and implementation model	System integration projects	Software deployment
Rajala and Westerlund (2007)	Generic types of models	Models oriented to close cooperation with the customer, offer of tailor-made solutions (high, low)	Models using a standard IT platform for building customer solutions (high, high)
	Degree of involvement	High	High
	Level of homogeneity	Low	High
Popp (2011)	Type of goods or services	Intangible goods (software and intellectual property)	Intangible goods (software and intellectual property)
	The business model archetype	Distributor	Distributor
	The revenue model	Direct/commission	Direct
Wartini-Twardowska, 2014	Position in the value chain of the software industry	Implementation	Distribution
	Basic strategies for creating added value	Close integration with the customer	Close integration with the customer

**Table 3:** Characteristics of the most differentiated INT and VAR models using the Software Business Model Framework.

Item	Characteristics	Generic Model: INT	Generic Model: VAR
1	STRATEGY		
1.1	Value Proposition	Intimate Customer Relationship	Functionality; Quality
1.2	Value Chain	Implementation, support	Implementation, maintenance
1.3	Degree of Vertical Integration	High	Low
1.4	# of Cooperation Partners	Many	Few
2	REVENUE		
2.1	Revenue Source	Direct/Commission	Direct
3	UPSTREAM		
3.1	Software Stack Layer	All	Application Software
3.2	Degree of Standardization	Individual Production	Bulk Production
3.3	Key Cost Driver	Subcontracting	Third Party Software Licenses
4	DOWNSTREAM		
4.1	Target Customer	Large Organizations	All Organizations
4.2	Channels	Sales Agents, Events	Sales Agents; Telesales
5	USAGE		
5.1	Implementation Effort	High/Medium	Medium/Low
5.2	Support Model	Customer Specific Support	Standard Support

Source: Authors' analysis based on Schief and Buxmann (2012).

### 2.1 Business models and their typologies

According to Wirtz et al. (2016), from the 10 thematic groups of research on the essence of business models, covering: (1) operations; (2) implementation; (3) definitions and scope; (4) actors and interactions; (5) value systems; (6) forms and components; (7) design; (8) performance and controlling; (9) change and evolution; and (10) innovation; most of these (groups (1) to (6)) are still scarcely analyzed. The least frequently tackled issues include operations and implementation of business models, followed by business model definitions and scope. Actors and interactions are also rarely studied, while the state of research in the next three areas, the value system, forms and components of a business model, as well as design (Wirtz et al., 2016), is slightly better (although also scant). According to Qiao et al. (2017), business model innovation and value-added creation have become the main research topics around business models. Most authors define the business model by focusing on its general structure and various components or sub-models (e.g., Fjeldstad and Snow, 2017; Wirtz et al., 2016; Osterwalder and Pigneur, 2011; Wirtz, 2011; Demil and Lecocq, 2010; Chesbrough and Rosenbloom, 2002). Wirtz et al. (2016) point out that there are other methods for defining business models, such as: descriptions (Afuah and Tucci, 2003), reference frameworks (Eriksson and Penker, 2000), architecture (Linder and Cantrell, 2000; Timmers, 1998), and configuration (Baden-Fuller and Mangematin, 2013). There is a consensus in the literature regarding the most important function of the business model, namely, as a simplified and aggregated representation of business architecture (Wirtz et al., 2016). Understanding and classifying the key features (variables) of the business model and linking them to specific results are frequent research problems in this field (Eckhardt, 2013). Our research takes us one step further. The classified variables are closely related to the type of business model. The research conducted thus far proves that the typology of the business model enables the possibility of configuring the business architecture of an organization in such a way as to cross the boundaries of time, industry, or traditional business (Baden-Fuller and Mangematin, 2013).

The majority of the experts who participated in the study conducted by Wirtz et al. (2016) attributed great importance to interactions between: components of a business model (see Hamel, 2000); business models at the company level or complex business model forms (company groups) (see Wartini-Twardowska, 2014; Markides, 2008; Markides and Charitou, 2004); or business models at the industry level (see Xu et al., 2017). In this context, a network-oriented view is identified in literature as well (Jabłoński, 2015). Researchers, therefore, recognize the importance of the industry environment in the development of business models (see Lu and Ramamurthy, 2010).

Our research is done in the software industry environment. The context of business architecture design in the software industry can be described as follows: (1) progressive concentration of the industry—significant M&As among companies; (2) new technologies—the implementation of an ERP system, even the most advanced one, is no longer a source of competitive advantage (Carr, 2004); (3) increased importance of the service model as opposed to the product model (Suarez et al., 2013); (4) the growing importance of shared service centers, outsourcing of IT services, and cloud computing; (5) mobile access to information—the possibility of making decisions in real time; (6) open source software development; and (7) relatively easy access to capital (e.g., seed capital/venture capital). Such a defined context forces serious changes in the business models of technology companies (see Fichman, 2004). Knowledge of the types of business models used by companies operating in the software industry is, in our opinion, the starting point of many analyses, including those devoted to value creation. Thus far, the most important results of research on business models in software industry companies include: (a) classifications and characteristics of the business models (e.g., Schief, 2014; Wartini-Twardowska, 2014; Buxmann et al., 2013); (b) interactions between components of the business model (Casadesus-Masanell and Ricart, 2010); (c) design of the business models (Casadesus-Masanell & Llanes, 2009); (d) performance measurement at the business unit level (Samsonowa, 2011); (e) business models of company groups in the context of the consolidation of financial transaction effects; and (f) business model innovations (Rajala et al., 2012; Van Putten and Schief, 2012; Johnson et al., 1996). Li et al. (2010) found that higher operational capacity increased the survival of software companies more than higher marketing and R&D opportunities. We believe, the application of an additional approach based on the typology of business models in the software industry would enable a stronger justification of the research results.

Accordingly, in the subsequent sections, we develop an approach that includes a description of the INT and VAR business models. Our study reveals a picture of a business model combination (INT-VAR) and its potential benefits (market/operational/development processes). As the synergy potential will vary depending on the types of business models that are combined, we believe this is a gap in the extant research that needs to be urgently addressed.

### 2.2 State of the research on synergies from a business combinations

The study of M&As has been a stream in the company finance, economics, strategic management, and organizational behavior literature for decades (Zollo and Meier, 2008; Mukherjee et al., 2004; Larsson and Finkelstein, 1999). Despite the huge number of studies carried out, there is a wide divergence of opinions on how synergies should be measured. Existing approaches range from subjective (e.g., qualitative assessments of degrees of synergy realization; integration processes; synergy pitfall reduction) to objective measurement methodologies (e.g., financial), and from organizational level to industry level analyses (see Table 4). Synergy is the main factor in value creation in M&As and the most important motive behind them as well. However, synergistic effects are difficult to achieve and among the reasons are the synergy pitfalls that threaten them (Fiorentino and Garzella, 2015). According to Sirower (1997), the synergy trap occurs among companies involved in large acquisitions that have not made pre-acquisition plans. However, even for the 2 out of 10 that do plan for performance improvements required in the pre-acquisition price, the uncertainty of competitor reactions can limit synergies (Sirower, 1997). The M&A process is a fast growth strategy frequently implemented by high-tech companies (Mchawrab, 2016). Yet, this growth through M&A comprises relatively higher risk. The reason acquisitions fail, according to Mchawrab (2016), is the fault of the valuation methods and tools that are not adapted to the specifics of the industry.

**Table 4:** Benefits of synergy in selected studies on M & As.

Author	Approach/method/ model/procedure/tool	Context/Debate	Characteristics of synergy/Ways of manifesting synergy	Findings in the context of synergy
Ansoff (1965)	• The measurement of synergy as a contributing factor to potential joint profitability; • Hierarchy of (economic and noneconomic) objectives of an industry; • Model for diversification and/or expansion by making qualitative decisions; • The framework for measuring synergy of a new product-market entry; • Procedure for individual project evaluation and selection.	• M&As or internal growth, or both decisions; • Synergy as the primary variable in discussion of diversification strategy; • Synergy as a fourth component of strategy: (1) product-market scope; (2) growth vector; (3) competitive advantage; (4) synergy	• Start-up synergy: near-term profitability of the new entrants in the market; • Operating synergy for the industry: long-term profitability; • Expansion of present sales; • New product-markets	• Measuring the potential synergy and enhancing performance refine the assessment of an industry; • M&As are preferred over internal development when: - start-up synergy is weak and operating synergy is strong (unrelated horizontal and vertical diversification to apply); - start-up and operating synergies are weak or close to zero (concentric diversification should be applied)*; - start-up and operating synergies are close to zero (conglomerate diversification should be applied)*. • M & As are preferred alongside internal development. ∗ Exceptions to acquisitions (1) timing of no importance; (2) incipient demand; (3) unavailability of competent companies; (4) high price/earnings
Gruca et al. (1997)	• Case studies; • Micro-level phenomenon (the role played by the constituent parts).	Sharing relationships between and within business units	The sharing of resources across business activities is the basis for the synergy, but this may not be a sufficient condition for synergy because: • the resources being shared may not have the potential for synergy; • the potential synergy is not fully realized; • realized synergy itself may not always lead to sustainable competitive advantage. Form of synergies: • investment synergy; • customer (sales synergy); • raw materials (operating synergy); • management skills (management synergy**).**	Six stages in moving from resource sharing to sustained competitive advantage: (1) Shared resources must be critical to the value chain of the organization; (2) Shared resources must exhibit flexibility for there to be the potential for the synergy; (3) Sharing of critical resources should not come up against capacity constraints, there can be both an upper and a lower limit to sharing; (4) Costs of co-ordination can inhibit the realization of potential synergy; (5) The sharing must be unique and non-inimitable.
Larsson and Finkelstein (1999)	• The integrative model: synergy realization is a function of the similarity and complementarity of the two merging businesses (combination potential), the extent of interaction and co-ordination during the organizational integration process, and the lack of employee resistance to the combined entity; • Sample = 61 cases of M&As • Acquisition size based on: − “critical mass" argument; − "managerial attention".	Mechanisms of M&As	The degree of synergy realization	• The potential value-added of acquisition integration is somewhat muted when acquisitions have little synergy potential; • The acquisition integration is particularly important for realizing synergies when the combination potential is high; • Complementarities may well be an underappreciated source of value creation in M&As, acting both to boost synergy realization and to ease employee resistance.
Mukherjee et al. (2004)	The survey consisting of 23 questions	• Practices in M & As; • The number and average size; • Motives of M&As, including synergies; • Methods used to value the acquired; • Divestitures; • Agreement or disagreement with nine statements involving M&As.	Discounted cash flow	• Large companies become larger through repeated acquisitions of relatively large companies; • The synergy is the most important top-ranked motivation for M&As, and operating economies are the general source of synergy; • The valuation techniques: the DCF models as the primary method, and then the DCF models plus the market multiple method; • Three divestiture types and five motives; • Diversification protects during economic downturns; • Hostile acquisition often results in higher payment for those acquired.
Damodaran (2006), 2012	Three approaches to valuation: (1) DCF models; (2) Relative valuation; (3) Contingent claim valuation. Basic techniques for evaluating capital structure	Valuation tools	• Operating synergies: economics of scale; increased pricing power; higher growth potential; • Financial synergies: tax benefits; diversification; a higher debt capacity, and uses for excess cash; higher cash flows; lower discount rates	• The estimate of the value of synergy in three steps; • Errors in the valuation of synergy (e.g., wrong discount rate; wrong multiple for cash earnings); • Overpaying on acquisitions as one manifestation of poor management.
Chadam and Pastuszak (2013)	• Direct interviews with the CEO, CFO, and COOs (between the year 2000 and 2010 in several companies conducting acquisitions); • Sample = 132 cases of M&As; • Five sectors: production; building; gas and energy; IT and telecommunications; other services; • The process approach	The valuation of the real synergy	Different forms of the operation synergy: economies of scale; economies of scope; economies of competitive position	Identification of key conditions for success at each stage of the transaction process (critical success factors (CSF)), such as: • The price < the value of the expected synergy; • Well-planned acquisitions with high premiums fail; • Professional integration and the efficient and effective management of the new company after the integration.
Garzella and Fiorentino (2014)	• Questionnaire survey; • Interviews with M&A experts	The success of pre-acquisition planning	Three main approaches to synergy categorization based on: (1) Anglo-Saxon approach: cost-saving expectations and revenue growth opportunities; (2) European approach: a managerial perspective (e.g., operating synergies; financial synergies; tax synergies; increased market power); (3) Mixed approach.	Several mismatches in synergy measurement practices (e.g., strategic factors not adequately quantified). A synergy measurement model integrates the four following strategic factors: • Type of synergy; • Size of synergy; • Timing of synergy; • Likelihood of achievement.
Fiorentino and Garzella (2015)	A framework integrating the compatible elements of research and the main findings of studies on synergy	Synergy, as an important motivation for M&As, has tended to be overestimated and has been difficult to achieve	Relationships between several values of synergy	The dimensions of synergy pitfall management: (1) The steps of the M&A process; (2) Several values of synergy; (3) The effects of poor synergy management; (4) The potential causes of synergy inflation; (5) The selection of solutions to synergy pitfalls. Three relevant synergy pitfalls: (1) The “mirage” - a tendency to overestimate synergy potential; (2) The “gravity hill” - the underestimation of the difficulties in synergy realization; (3) “Amnesia” - a dangerous lack of attention to the realization of synergy.
Caiazza and Volpe (2015)	Based on: • Previous studies (Brocke & Sinnl, 2011; Gogan et al., 2013; Soni & Kodali, 2011); • Structured literature review	Cross-border M&As	Low level of absorptive capability leads companies to work in a common way but without any synergy	Three stages of the M&A process (1) Multilevel due-diligence; negotiation; closing (2) Organizational, cultural integration; (3) Atmosphere, distinctive competences. The challenges by cross-border M&As from strategic, cultural, organizational, and financial perspectives: • country level: e.g., different policies on foreign investment; conflicting antitrust requirements; • industry level: e.g., technological intensity; advertising intensity; • company level: e.g., differences in values, religions and behaviors; businesses specifically.

To provide a framework that enables a better understanding of the business and the sources of added value creation, we extended our research to software industry business models to discover the opportunities for synergy benefits from combining them. Such analysis is critical as synergy is the key to M&A survival and prosperity (Harris, 1981). To generate synergy, the acquirers must be able to: (1) limit competitors' abilities to contest current input markets, processes, or output markets, and (2) open new markets where these competitors cannot respond (Sirower, 1997). A common measure of M&A success is the increased value of the combined companies through existing value creation (Mukherjee et al., 2004). Many publications stress that the failure of the M&A is mainly due to overpaying for synergy (Damodaran, 2012; Johnson, 2000; Copeland et al., 1997). The value perceived by the acquirer and the acquired depends on the type of synergies possible and who can use them (Copeland et al., 1997). Synergies contain potential benefits the buyers are willing to pay for in whole or in part (Johnson, 2000). Admittedly, scholars categorize synergies in many ways (Garzella and Fiorentino, 2014; Kaplan, 2010; Thompson and Martin, 2005; Gruca et al., 1997). Cullinan et al. (2004), analyzing three types of cost synergies and two types of revenue synergies, omitted evaluating the acquisition price in the former categories. Contrary to this, KPMG (2011) recognized revenue synergies as the first important area of value growth from M&As. The most rarely undertaken research on synergies is in the context of the business model.

According to Markides (2008), integrating different business models will be successful if you look for ways to use synergies, no matter how small or limited they are. However, to increase the success of M&As, the potential acquirer should first assign the planned acquisition to one of five proposed integration categories and then consider the general obstacles to the integration of the acquisition (Chatterjee and Brueller, 2015). Christensen et al. (2011) use two approaches to integrate business models. As stated by Lovallo et al. (2008), poorly estimated synergies at the stage preceding the business combination may cause serious losses in the M&A. Therefore, a thorough analysis of M&A transactions in the context of synergy is an important use of time as it helps management avoid making the wrong choice. The methodology used here for measuring and assessing group synergy potential is a proven proposition that can support such M&A decisions.

## 3 Methodology

### 3.1 Research stages

The systematic search for sources and methods to assess the synergistic effects of M&A processes among groups comprising various business models requires the formulation of several key research questions. The first two research questions concern the description of the group's business architecture. Question one is concerned with the structure and interactions between the variables of the individual models.

**RQ1: How can the business architecture deriving from different business models be described using key variables and their interactions?**

The second question concerns the importance of the variables of the individual business models from the point of view of the design of the business architecture.

**RQ2: How can the business model variables be classified in terms of their importance in creating synergy potential through the combination of the models?**

The answers to these questions are important because the description of the structure of variables and quantification of their values allows the use of formal methods in the subsequent stages of the research, which, in turn, allows a better understanding of their importance in every business model. In looking for synergies in the M&A processes, the importance of the variables in one model must also be recognized for the implementation of the strategic objectives of the second model. This sets the stage for the subsequent research, which is to identify areas of potential opportunities and threats related to the creation of a business architecture consisting of various models, and the starting point to answer the third research question:

**RQ3: How can potential areas of opportunities and threats be identified for the planned combination of heterogeneous business models?**

The full assessment of the M&A effects requires not only the identification of opportunities and threats, but also the assessment of the contribution of individual models to the value of the business architecture being created. Such a contribution should be treated as a special parameter (weight) in the M&A risk assessment process. Thus, the last research question is:

**RQ4: How can the contribution of individual business models be measured to assess synergy potential of the business model combination?**

**Fig. 1:**
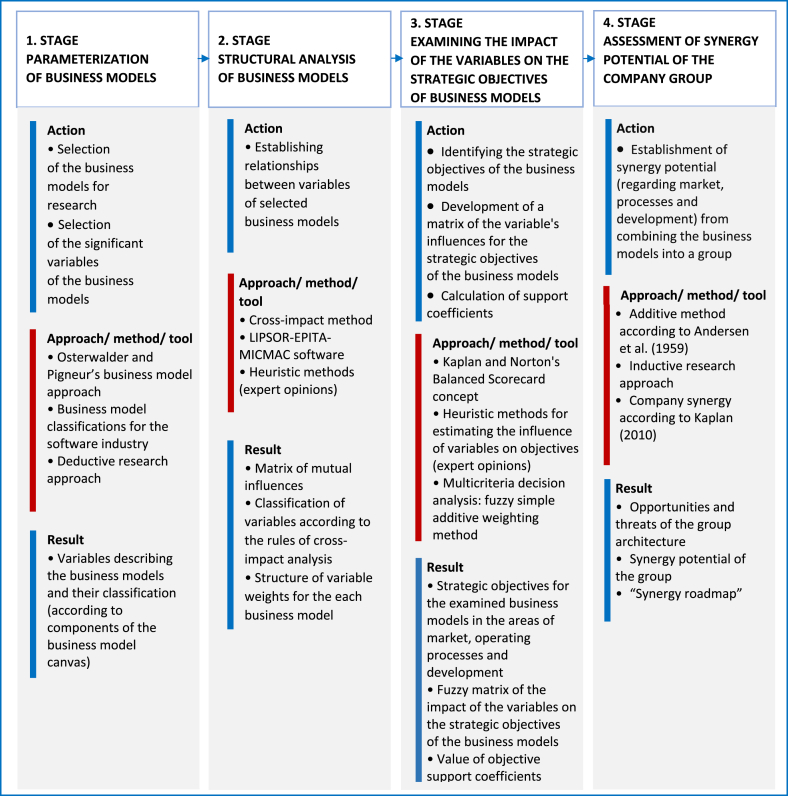
Research methodology for discovering synergy potential by combining business models.

To answer these research questions, we propose a special methodology covering four research stages (Fig. 1). As shown in Fig. 1, actions, methods, approaches, and tools are defined in each stage. The last column describes the expected results. The objective of the first stage is to define a business architecture that includes two of the generic business models in the software industry and their parameterization in terms of examining the synergy effects resulting from their combination. We selected two business models for further work, calling them, INT and VAR, respectively (see Tables 2 and 3). These models are characterized by a similar strategy of cooperation with the customer but a different position in the value chain of the software industry. The basic characteristics of the INT and VAR models, in light of various classifications of software industry business models (see Table 1), are presented in Tables 2 and 3. The detailed characteristics of the variables of the analyzed business models are discussed later in this paper.

We assume *a priori* that obtaining positive results from a business architecture consisting of two models will allow us to explore the synergy effects of any combination of business models. The parameterization of business models, as part of the first stage of the methodology, consists mainly of identifying the variables that best describe the properties of business models under study. Considering the research objectives, the quality of the results, and acquisition costs, we limited the maximum number of variables for each model to 40. When selecting variables, we used the list of variables defined in the research regarding the classification of business models in the software industry (Wartini-Twardowska, 2014). For each component of the Osterwalder and Pigneur canvas, we chose the minimum number of variables that best characterize the business model to fit within the accepted constraints. Next, we selected a dozen companies from the SME sector that met the criteria specified by the VAR and INT models to evaluate the variable values. The process of collecting data, including the selection of companies, is described in the “data collection” section.

The second stage of the research focuses on the structural analysis of the models using cross-impact analysis (section 3.3). The LIPSOR-EPITA-MICMAC software was used, and the structures of the variables obtained for each model allowed their quantification (i.e., the assignment of weights for each variable category). The weights of the variables were determined using the heuristic method, guided by the opinions of experts and the strategic objectives of the models; we subsequently used them in the next stage of the research, the fundamental stage. An additional description of the activities undertaken in this stage, including the structural analysis procedures, is detailed here.

This main research stage is the analysis of the impact of the variables of one model on the strategic objectives of the other. First, strategic objectives are defined for the business models under consideration, in line with Kaplan and Norton's Balanced Scorecard approach. Then, we applied the simple additive weighting (SAW) method—the most popular method of multi-criteria decision making. One particular advantage of this method is its intuitiveness in modeling the decision-maker's preferences. In addition, the variables (in the considered context: criteria) of the models must meet the necessary and sufficient condition to use the additive utility function. The preferential independence of the criteria is fulfilled (i.e., both criteria are independent in a preferential sense, if the decision-maker's preference for one is independent of the assessment in relation to the other). Thus, the SAW is used as the starting point for the impact analysis (Churchman and Ackoff, 1954); however, the results are not satisfactory due to large discrepancies in the experts' opinions regarding the estimated values of inflows. Next, we changed the SAW method to the fuzzy simple additive weighting (FSAW) method (Tzeng and Huang, 2011). In this analysis, the most difficult element is always the rationale for choosing the value of the criteria weights and the strength of the mutual interactions. Considering the inaccuracies in estimating weights and the expert opinions allows this problem to be largely solved (Targiel, 2014). Ultimately, the proposed approach uses fuzzy expert opinions regarding the impacts of variables on the objectives of the model and the sharp (precise) values of weights for the model variables. Knowing the strength of the impact of one model on the individual strategic objectives of the other we identify the opportunities and threats to achieving the individual objectives of the considered models and the created business architecture (section 4.1). We finalize our research by assessing the contribution of each of the models in the M&A in creating value in the new business architecture (section 4.2). The aim of adopting Kaplan and Norton's Balanced Scorecard approach is to identify synergies according to the four perspectives of the BSC (Kaplan, 2010; Kaplan and Norton, 2001). We limit our study to three perspectives, excluding financial synergies. Financial synergies require the use of quantitative data (capital, cash flow, and profits from combining companies) and, thus, have to be refered to specific combination cases. First, we determine the potential opportunities and threats inherent in the combined company from the perspective of the market, operating processes, and development. Then, we reveal the synergy of each model. Next, we determine the consolidated impacts of the key business model variables on the group objectives. We finalize our research with the presentation of the synergy potential for this newly built business architecture. Assessing the contribution of combining business models into a group added value requires a simultaneous approach that uses the consolidated data of single-level groups. We apply the additive method to understand the consolidated values of the influences of the variables in achieving the objectives of the group.

### 3.2 Data collection

The basic source of data was direct interviews with representatives of companies and experts from the software industry, preceded by the analysis of websites selected for the survey of companies. A step by step procedure was used, which consisted of verifying the partial results obtained from the expert opinions. Based on previous experience related to the classification of business models for the software industry, the authors developed a dedicated questionnaire that was answered by conducting interviews with representatives of selected companies. The selection was preceded by consultations with experts, i.e., experienced representatives of economic practice. The companies selected for the research were SMEs operating in one country in the software industry. Ultimately, the selection criteria were best met by 10 (VAR) companies and 5 (INT) companies. The results of the questionnaire were examined by both experts and the representatives of selected companies. In the case of significant differences in assessment, additional interviews were conducted to explain the reasons for the discrepancy in the results obtained. Experts assessed the interactions between variables based on professional experience and knowledge of the companies representing the business models under study.

### 3.3 Data analysis: structural analysis of business models

By the proposed methodology, the purpose of the structural analysis of the business models is to identify and classify variables that characterize the business models. In this way we search for answers to the first research question (RQ1). The procedure for such an analysis includes four following steps.

Step 1: Identification of relevant model variables

The variables for the models were set based on Osterwalder and Pigneur‘s business model approach. For each component, based on the relevant literature and the experience of the cooperating experts, we proposed a set of variables, which was then subjected to initial verification by a pilot group of representatives of selected companies from the software industry. The results of the data analysis finally allowed 40 variables for each business model to be obtained.

Step 2: Analysis of mutual influences

The selected variables were the basis for the development of the so-called Influence matrix. The variables for each model were placed in rows and columns to form a 40 × 40 matrix. The matrix was filled with the experts' opinions according to the following rules: if the first variable has no effect on the second variable, then 0 is entered into the matrix, if the impact is identified, its strength is evaluated on a scale from weak (1) to moderate (2) to strong (3). The data was then processed by the LIPSOR-EPITA-MICMAC software, where we received the classification of variables together with the graphical visualization of mutual influences for each matrix of the two business models. The results obtained were then analyzed from the point of view of the significance of particular variables for each business model.

Steps 3 and 4: Classification and quantification of variables

**Fig. 2:**
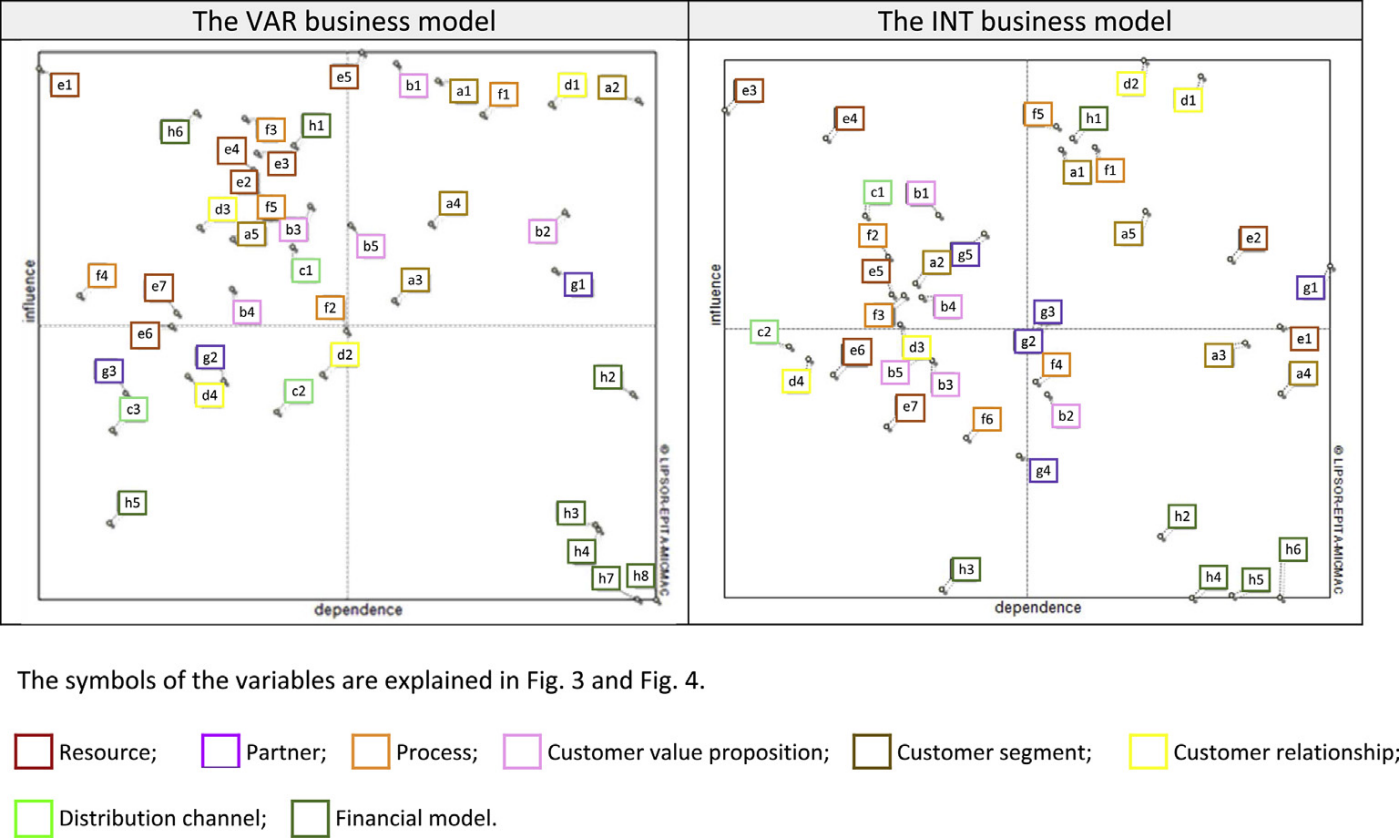
Indirect influence/dependence map for the VAR and INT business models.

**Fig. 3:**
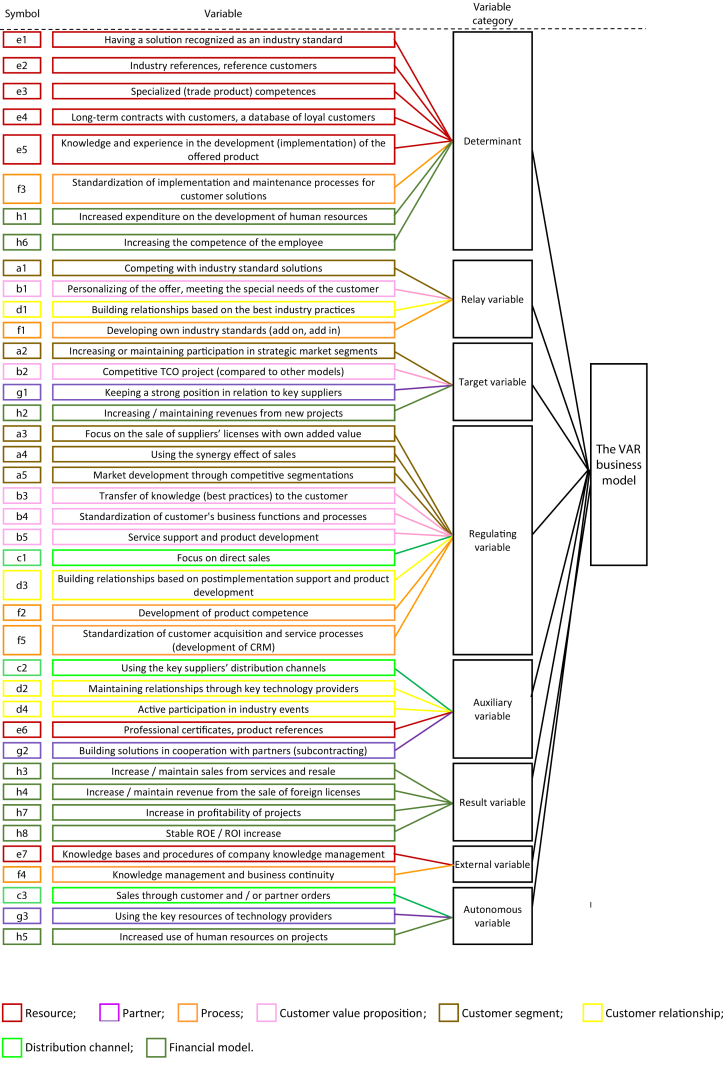
Business model structure of the VAR.

**Fig. 4:**
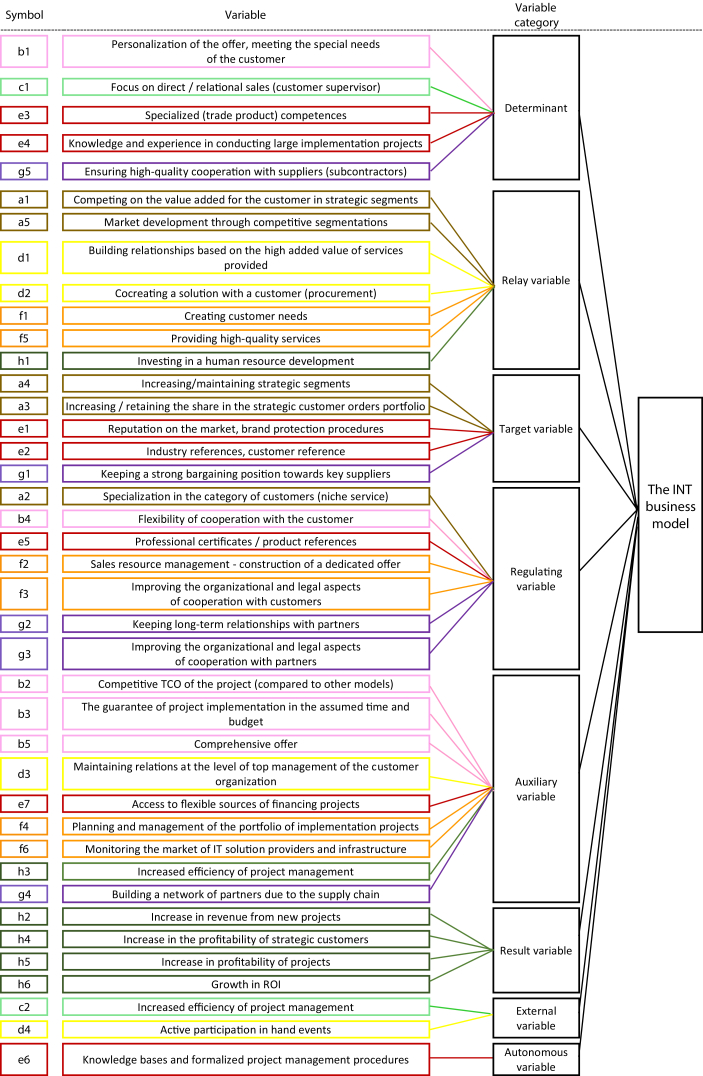
Business model structure of the INT.

The interpretation of the results of the analysis was based on the classification of variables using the criterion of influence and dependence (Fig. 2). The identified variables were classified into the eight categories presented in Fig. 3 and Fig. 4.

The identified variables were qualified into eight categories according to the following rules (see Arcade et al.): (1) determinants – strong influence, weak dependence; (2) relay variables – strong influence, strong dependence; (3) target variables – moderate influence, strong dependence; (4) regulating variable – moderate influence, moderate dependence; (5) auxiliary variables – weak influence, moderate dependence; (6) result variables – weak influence, strong dependence; (7) external variables – moderate influence, weak dependence; and (8) autonomous variables – weak influence, weak dependence. Then, the variables were quantified using the FSAW method, which was adopted in the impact analysis of variables of one model on the strategic objectives of the other model. The starting point was the acceptance of variable weights, where 50% corresponds to the sum of the determinants and relay variables. The structural analysis was performed on a sample of the most popular business models of the Polish IT companies - the INT and the VAR.

Parameterization of the models showed that the VAR is a more complex business model than the INT because it has a larger number of variables between which the strongest or strong interactions occur (see Fig. 2). The determinants of the VAR come primarily from the key resources area (variables “e”). The other determinants are the variables describing the key process (variable “f”) and the financial model (variable “h”) (see Fig. 3). The determinants of the INT, in addition to resources (variable “e”), also include acquisition oriented variables and specialist customer service (value proposition variable “b” and distribution channel variable “c”) as well as selected aspects of cooperation with subcontractors (business partner variable “g”) (Fig. 4).

The relay variables of the VAR determine the competitive strategy of this model in the software industry (variables of customer segments "a" and processes "f") (see Fig. 3). The next two variables shape the value proposition for the customer (the variables of the value proposition "b" and customer relationships "d"). The same applies to the INT. In addition to the competition strategy in the industry, which describes the variables of customer segments "a" and processes "f", there are also variables characterizing the creation of value propositions for customers (variables of relationships with customers "d"), as well as the financial model (variable h) (see Fig. 4).

The common target variable for the VAR and the INT is to increase share in key market segments (customer segments variables “a”). The other objectives of the models result directly from various strategies of competing in segments (see Fig. 3; Fig. 4).

The VAR and INT regulating variables include, in particular, variables responsible for achieving market objectives (variables of customer segments "a"), as well as for shaping customer value propositions (variables "b" and variables of processes “f ") (Fig.3 and Fig. 4). VAR regulating variables also describe distribution channels (variable "c") and customer relationships (variable "d") (see Fig. 3). The variables regulating the INT are also characterized by resources (variable "e") and cooperation with subcontractors (variables g) (see Fig. 4).

Due to the relatively weak impact on other variables and moderate vulnerability, their importance for the model can be considered only in the context of other variables. The auxiliary variables are listed in Fig. 3 and Fig. 4.

The result variables are selected variables of the financial model ("h"), which are good early warning indicators (see Fig. 3; Fig. 4).

From among the variables examined, the authors selected the same number of external variables for both business models, but belonging to different groups (cf., Fig. 3; Fig. 4).

The VAR model is described by a larger number of autonomous variables, which describe in turn: distribution channels (variable “c”), cooperation with subcontractors (variable “g”) and financial model (variable “h”) (Fig. 3). One only variable from the resource area ("e"), an autonomous variable, is characterized in the INT (Fig. 4).

Each of the eight variable categories of one business model (INT/VAR) has a diversified impact on the objectives of the second business model (VAR/INT), which in the case of a combination decision generates specific results that are presented in the next section.

## 4 Results

The fourth section contains both the characteristics of the proposed approach to identifying the impact of variables on the strategic objectives of business models as well as the assessment of contribution of each business model to the synergy potential of company group. In this section the answers have been given to further three research questions (RQ2, RQ3 and RQ4).

### 4.1 Impact of variables on the strategic objectives of the business models

#### 4.1.1 Proposed approach

The strategic objectives for the analyzed business models are based on the concept of Kaplan and Norton‘s Balanced Scorecard. The objectives were formulated at a relatively high level of generality to be consistent with the objectives of as many of the organizations participating in the research as possible. In three areas of our investigations, i.e., market, operating processes and development, four strategic objectives have been defined for each model. Then, using the FSAW method, the impacts of the variables on the strategic objectives of each model were identified. The analysis of the strength of such inflows allowed for the benefits and risks of combining the business models to be better estimated. Section 4 presents the most important variables, the impact of which was at least 7%, and together with the other most important variables formed the majority of the total impact on three separate categories of objectives (market, operating processes and development).

#### 4.1.2 Market objectives

Both business models are beneficiaries in this combination (see Table 5). The potential benefits of VAR are higher, but on the other hand, they are associated with greater risk, as demonstrated by the analysis of opportunities and threats. For the VAR, the highest opportunity for integration is access to the markets of the INT. However, the potential combination is provided by the INT increasing the share in segments and orders of strategic customers, by offering a new product/service (target variables a4 and a3; Fig. 4). The common objective for both models is to increase the share in key market segments in each model. The other model objectives are directly derived from the various strategies of competing in the segments. An in-depth analysis of the impact of variables on the objectives of business models allows us to identify variables that determine the increase in value for each model.

**Table 5:** A comparison of the impact value of the INT and the VAR on the market objectives and operating process objectives of the VAR and the INT.

**Market objectives**					
Business model of the INT			Business model of the VAR		
The objectives of VAR			The objectives of INT		
1.1	1.1.1 Increased share in current market segments	2.17	2.32	1.1.1 Increased share in segments and orders of strategic customers	1.1
	1.1.2 Developing new markets	2.11	1.60	1.1.2 Maintaining long-term relationships with strategic customers	
1.2	1.2.1 Improving customer segmentation processes	1.37	2.07	1.2.1 Developing an offer of new services in current markets	1.2
	1.2.2 Developing postsales services	2.04	1.16	1.2.2 The developing of the offer of standard services in new markets	
	**Total**	**7.69**	**7.15**	**Total**	
**Operating process objectives**					
Business model of the INT			Business model of the VAR		
Objectives of the VAR			Objectives of the INT		
2.1	2.1.1 Standardization of the application's business functions and processes	1.62	1.14	2.1.1 Improving the processes of early recognition of customer needs	2.1
	2.1.2 Knowledge management and business continuity/development of applications and technologies	1.30	1.37	2.1.2 Improvement of after-sales service of customers	
2.2	2.2.1 Development of acquisition automation processes and customer service	0.89	1.51	2.2.1 Building a balanced portfolio of projects	2.2
	2.2.2 Improvement of service processes, change management and incidents	1.80	1.49	2.2.2 Maintaining a dominant bargaining position towards subcontractors	
	**Total**	**5.61**	**5.51**	**Total**	

##### 4.1.2.1 Market objectives of the VAR

**Fig. 5:**
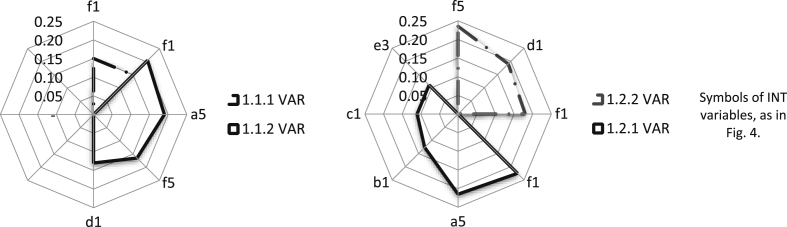
The INT variable group with a dominant influence on market objectives 1.1.1, 1.1.2, 1.2.1, and 1.2.2 of the VAR.

The increase in the market segment share, through combination with the INT, is the fastest if both companies support the same or very similar segments. The INT becomes a specific distribution channel for solutions of the VAR. INT can offer VAR products on its markets, e.g., as upselling (objective 1.1.1), as well as being able to create new markets for the VAR, offering value to the customer by integrating the solutions of many IT providers. VAR solutions become, in such a case, a component of a whole offered by INT under its brand. Therefore, the INT opens the VAR's access to markets that it would not be able to enter on its own (objective 1.1.2). As a result, the VAR can provide advanced maintenance services that support solutions offered by INT (objective 1.2.2). The objective 1.2.1 is slightly more difficult to achieve, which can be justified by the focus of the INT on large integration projects as opposed to the VAR's interest in the more massive sale of its products. We have identified seven variables of the INT that have a significant impact on the market objectives of the VAR. Among them, there is the relay variable f1, which supports all the VAR market objectives (see Fig. 5). The development of maintenance services (objective 1.2.2) is most strongly supported by the relay variables of the INT f5. The last of the VAR (1.2.1) objectives must be implemented by VAR regardless of the potential connection to the INT. VAR has to develop its own market segmentation procedures and distribution channels based on partner offerings, which should be treated as a complementary source of value creation. The most important opportunities and threats of creating a business architecture from the perspective of VAR create a synthetic image of the directions for combining business models (Table 6).

**Table 6:** Opportunities and threats for the business architecture of the VAR/INT from the perspective of the VAR/INT. Market objectives.

Market objectives	
Opportunity of the VAR	Threat of the VAR
• Access to market segments served by INT due to: - Potential opportunity to increase the attractiveness of the VAR offer by offering the value offered by the INT; - Using key variables of the INT: creating needs and specialized commercial competences;	• The sales process of the INT offer may not be adjusted to the sales specifics of the VAR product;
	• Specialized commercial competences of the INT may be an obstacle to the mass distribution of the VAR offer;
	• Relatively low cost of switching supplier for the INT, especially on attractive, highly competitive markets;
• Creating an effective distribution channel for VAR solutions within the INT offer on new markets - using key INT sales competences and focusing on direct sales;	• Creating new markets for INT solutions may not correspond to the development directions of the VAR offer;
• High value added of the services provided by the INT opens the market for specialized maintenance services. VAR competes with INT competences to ensure high-quality services.	• Very strong dependence of the VAR on distribution channels of the INT.
	• Demand for services of the INT, due to their nonstandard profile, may be difficult to meet by the VAR.
Opportunity of the INT	Treat of the INT
• The distribution of the personalized VAR offer in the markets of the INT is a new element of the value proposition that strengthens the INT position on its markets;	• The dependence of VAR on the directions of developing the value propositions of the main technology supplier. There is a serious risk of evolving VAR value propositions and developing competencies in a direction that does not correspond to the INT‘s offer;
• The distribution of a personalized VAR offer is a component of the solution offered by the INT in new and existing markets;	• Requirements of INT customers regarding too deep personalization of the VAR offer, which is very different from the developed industry standard;
• Access to market segments supported by the VAR - it gives the potential to increase the attractiveness of the VAR offer by the value provided by the INT.	• Limited sales potential of the VAR solution in new markets attractive for the INT. The best industry practices of the VAR may not meet the expectations of new markets;
	• Limited sales potential of the INT's services in the markets of the VAR. The sale of VAR products is strongly subordinated to specific market segments, which means that INT specialized commercial competences may not be used.

##### 4.1.2.2 Market objectives of the INT

**Fig. 6:**
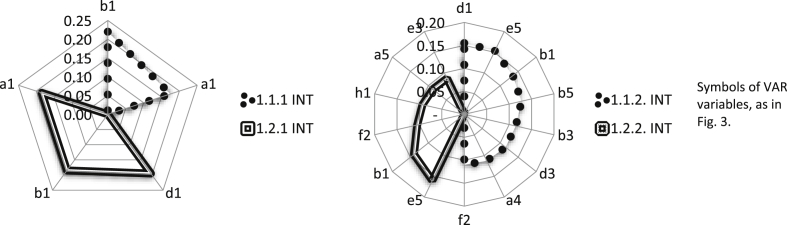
The VAR variable group with a dominant impact on market objectives 1.1.1, 1.1.2, 1.2.1, and 1.2.2 of the INT.

The crucial direction of the increase in INT share in its markets is to expand the product offer with new solutions that are attractive for the customer. Such a solution may be the value propositions offered by the VAR. The increase in the share in the segments and orders of strategic customers as the INT market objective (1.1.1) can, therefore, be treated as a higher-level objective in relation to objective 1.2.1 (Table 5). In turn, maintaining long-term relationships with customers can only be implemented to a small extent due to the VAR offer. Twelve variables of the VAR significantly affect the INT market objectives (see Fig. 6). Among them is the relay variable b1, which supports all the INT market objectives. INT can win long-term relationships with strategic customers (objective 1.1.2), in particular, due to customer relations (variable d1) and VAR resources (e5). The offer of standard products/services in new markets (objective 1.2.2) receives very strong support by the determinant e5 (Fig. 6). Such support is particularly important in the case of attempts to expand into new markets with an extraneous solution. A synthetic comparison of the directions of the business model combination gives the most important opportunities for and threats to the creation of such a business architecture from the INT perspective (Table 6).

#### 4.1.3 Operating process objectives

The total support for strategic objectives in the area of operating processes improvement is definitely weaker in relation to market objectives. The total impact of variables on the strategic operating objectives of both models is similar (see Table 5).

##### 4.1.3.1 Operating processes objectives of the VAR

**Fig. 7:**
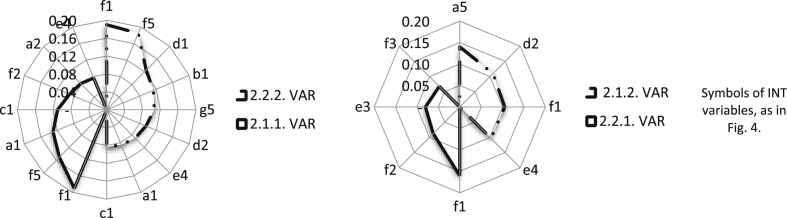
The INT variable group with a dominant influence on objectives 2.1.1, 2.1.2, 2.2.1, and 2.2.2 of the VAR in the area of operating processes.

The strongest support in the considered area was achieved by improving service processes, change management and incidents (VAR objective 2.2.2). A stabilization of receipts from maintenance services of the VAR allows for the safe planning of investments in product development. Fourteen variables of the INT have a significant impact on the VAR objectives. Relay variable f1, which is related to key INT processes, supports all operating process objectives of the VAR (see Fig. 7). The combination with the INT supports the management of knowledge and continuity of operations (objective 2.1.2), as well as the automation process development of acquiring and servicing customers (objective 2.2.1), much less (see Fig. 7). The most important opportunities and threats of creating a business architecture from the VAR perspective create a synthetic view of the directions of combining the business models (Table 7).

**Table 7:** Opportunities and threats for the business architecture of the VAR/INT from the perspective of the VAR/INT. Operating process objectives.

Operating process objectives	
Opportunity of the VAR	Threat of the VAR
• Development of own functions and operating processes is based on the needs created by INT customers and competitive market segmentations;	• The development of new VAR competences is almost always related to high investments involving the acquisition of new resources and often re-engineering of operating processes;
• The combination provides an opportunity to develop its own unique product competences and strengthen its competitive position in the reseller market;	• There may be big differences in the approach to quality management between VAR and INT resulting from, among others, the strategy of positioning companies in various market segments and organizational cultures;
• Combination with the INT enforces the application of standards of service quality management based on the best INT practices;	• The paradigm of cooperation with the customer based on, for example, presumption may require very deep changes in the structure of competences and resources of the VAR;
• Development of new implementation standards based on a different project management paradigm for particularly demanding customers;	• Sales resource focus on customers that require high value-added services can significantly reduce VAR competition using the industry standard. Having such a standard by VAR may lose its importance.
• Increasing the efficiency of customer acquisition processes due to the possibility of sharing resources with INT in the sales and marketing area.	
Opportunity of the INT	Threat of the INT
• The INT has a chance to strengthen its market position through stronger integration with the customer based on maintaining and developing a product by the VAR;	• The quality of the technology offered by the supplier has a very strong impact on the VAR's final product. The high cost of changing the main technology provider results from the developed VAR competences and often from sharing key resources with the main technology provider;
• The high added value of services provided by the INT requires, for each offer, an individual approach to customer expectations. Personalization of the offer based on its own industry standard of the VAR significantly supports the strategy of obtaining and postimplementation support of INT customers;	• The requirement of INT customers for far-reaching personalization offers is significantly different from the developed industry standard of the VAR;
• Providing a strong technological base in the form of VAR subcontractors allows achieving a strong position INT in relation to technology suppliers and other subcontractors.	• There is a risk of acquiring key customers of the VAR by the main technology provider for the VAR;
	• Liberty in shaping the pricing policy in the sale of products of the main VAR technology provider may be limited.

##### 4.1.3.2 Operating processes objectives of the INT

**Fig. 8:**
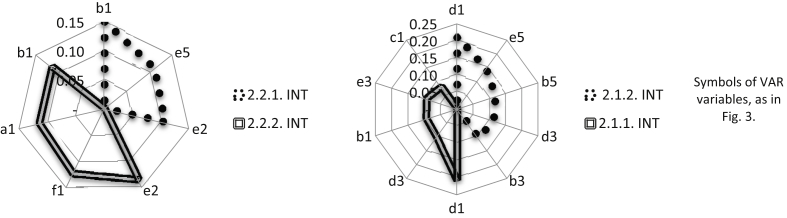
The VAR variable group with a dominant impact on the INT objectives 2.1.1, 2.1.2, 2.2.1, and 2.2.2 in the operating process area.

Expectations of the INT focus primarily on maintaining a balanced portfolio of projects (objective 2.2.1) and dominant position with respect to subcontractors (objective 2.2.2). The results of the analysis clearly indicate that the INT seeks to provide itself with a strong technological base (in the form of a network of subcontractors). The two objectives (2.2.1 and 2.2.2) of the INT are supported similarly by the variables of the VAR (Table 5). Therefore, the variable b1 (Fig. 8) is mainly used to balance the INT portfolio (objective 2.2.1), and variable e2 strengthens a dominant bargaining position towards subcontractors (objective 2.2.2). Building relationships based on the best industry practices (d1) of the VAR, from of all variables, has the strongest impact on objectives 2.1.2 and 2.1.1 (Fig. 8). The VAR competences in building relationships based on the best industry practices (d1) of the INT can help the INT successfully use both to improve its own processes of recognizing customer needs and after-sales support. A synthetic view of the directions of combining business models gives the most important opportunities for and threats to the creation of such a business architecture from the INT perspective (Table 7).

### 4.2 Potential contribution by combining business models to the value of a group

#### 4.2.1 Proposed approach

**Fig. 9:**
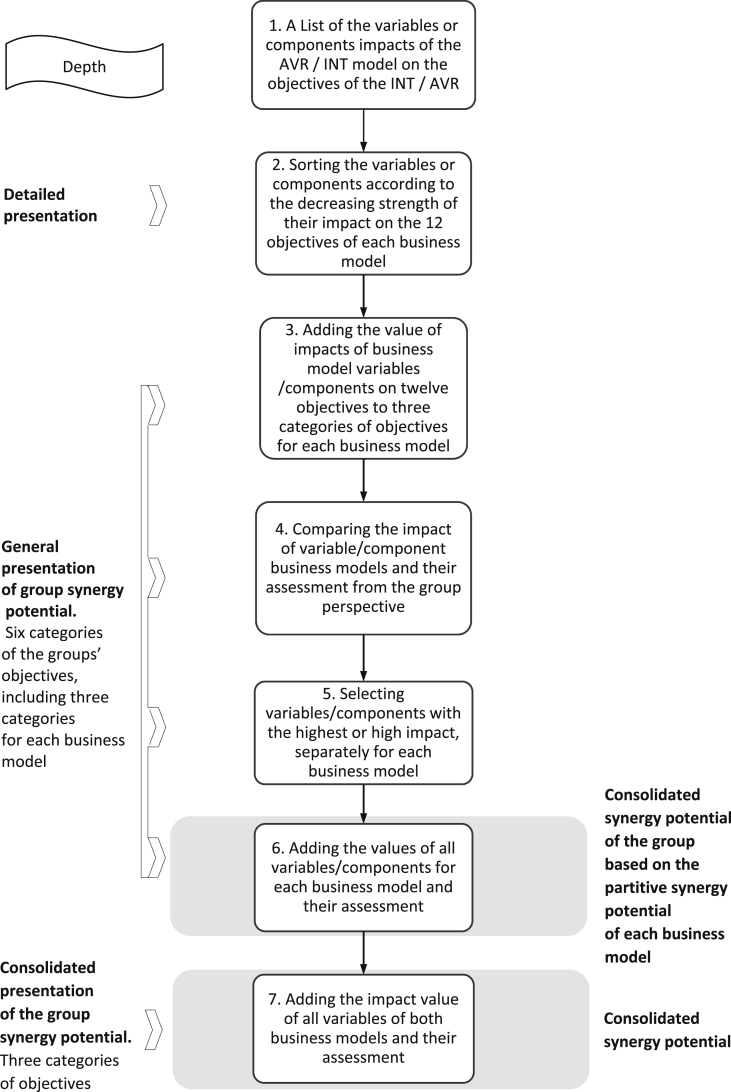
Assessment procedure of group synergy potential.

**Fig. 10:**
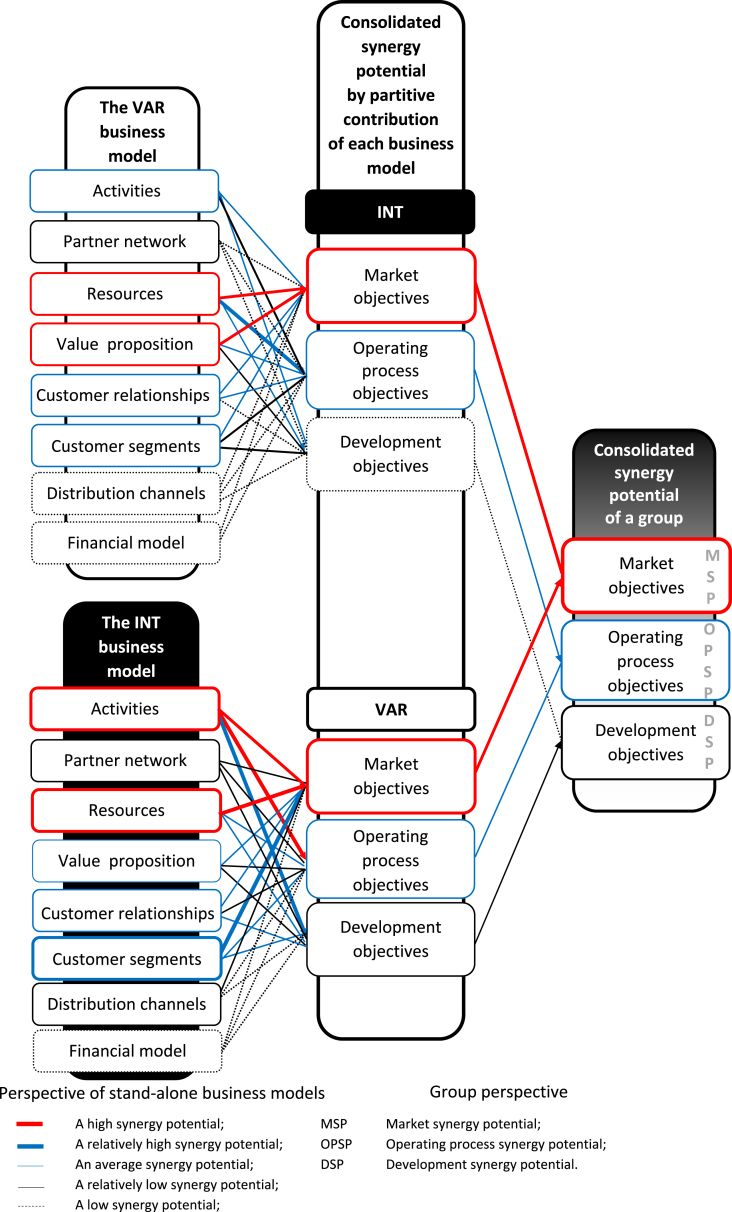
Synergy potential by two assessment perspectives.

The increase in the group added value, which consists of more than one business model, is assessed by its ability to generate synergies in three areas: market, operating processes and development. We understand the synergy potential of the group as an ability to support the business objectives of the group of companies by combining and sharing their markets, distribution channels, processes, resources, as well as transferring knowledge and learning. Thus, the final result of our research is to outline the real synergic potential for the planned business architecture of the group, the use of which will create added value. Fig. 9 shows the steps to determine the synergy potential of the group. In contrast, Fig. 10 illustrates the differing synergy potentials dependent on the assessment perspective. The assessment of the consolidated synergic potential of a group may differ significantly from the assessment made only from the perspective of a stand-alone business model. For example, key variables/components, considered from the viewpoint of the business model of the company forming the group, may lose their importance when considering the perspective of the business objectives of the group as a whole. However, identifying the potential of each business model is important because it allows their contribution to the consolidated added value of the group to be explained. This information cannot be obtained based only on consolidated values. A detailed and general presentation of the synergy potential of the group is based on the following scale of impact assessment of variables/components of the business models on the group objectives: 100%–80% – Strong influence; 80%–60% – Relatively strong influence; 60%–40% – Moderate influence; 40%–20% – Weak influence; 20% and less – Weakest influence. Finally, we selected variables of one business model (INT/VAR) that have at least a strong impact on the implementation of the objectives of the second business model (VAR/INT).

#### 4.2.2 Consolidated synergy potential of a group

The combination of the VAR and INT creates the high synergy potential only in the market objective area (Fig. 10). The average synergy potential concerns operating processes. The relatively low synergy potential is in the development area.

##### 4.2.2.1 Market objectives

The high synergy potential of the examinated combination is made by resources. Their importance is similar both from the perspective of the group and the stand-alone business models. The INT activities and customer value proposition of the VAR have a slightly lower importance for the group. The customer segments of the INT operate strongly to realize the market objectives of the VAR. The potential contribution by the rest of the components to the group added value is significantly lower. Among all VAR and INT variables, only three variables give the highest opportunities to use market opportunities to create the added value of this combination. At the same time, they are strong drivers of the success of the combination (Table 6).

##### 4.2.2.2 Operating process objectives

The impact of the VAR and INT variables in the implementation of the group operating objectives is weaker compared to their impact from the perspective of the stand-alone business model. Developing own industry standards and building relationships based on the best practices give the highest opportunities to use them for this combination (Table 7). The most important variables supporting the synergies from the combination of the VAR-INT belong to the relay and determining categories, which increase the chances of creating value for the group because they are at the center of attention of both business models. At the same time, they become an element of the "synergy roadmap" depicting actions to exploit the synergy potential (see Table 8).

**Table 8:** “Synergy roadmap” - reference VAR - INT group.

	Market synergy		Operating processes synergy	
Variable of a business model	VAR	INT	VAR	INT
f 1 INT	**Increase share in the current market and enter the new market by satisfying the needs of INT customers** *Develop a new offer of after-sales services by creating the new needs of INT customers*	—	**Improve the standardization of business functions/of processes in customer solutions and change (incident) management services by leveraging the INT capabilities to create customer needs**	—
b1 VAR	—	**Increase market share for strategic customer segments and offer new products through the use of the industry standard of the VAR**	—	*Develop automation processes to acquire and service customers through personalization of the offer based on the VAR industry standard*
e5 VAR	—	**Increase the share in the strategic customer segments and expand the offer in the existing markets by using the VAR competence in the field of the product development and implementation**	—	—
f5 INT	*Increase participation in the current market and make a new offer of after-sales services through greater opportunities to provide high-quality services*	—	—	—
a5 INT	*To gain a new market using customer segmentation of the INT*	—	—	—
d1 INT d1 VAR	*Increase the ability to sell after sales services based on high value added by the INT*	*Increase participation in strategic customer segments through the use of the best industry practices of the VAR*	—	**Improve after-sales service by disseminating industry-leading practices of the VAR**
c1 INT	*Use the INT distribution channels to gain new customers*		—	

Bold signifies that moves that strongly drive the added value;

Italic signifies that moves that relatively strongly drive the added value.

## 5 Discussion & conclusions

The identification of the most important model variables is of fundamental importance for the assessment of the risks associated with the creation of a business architecture based on different business models. The Osterwalder and Pigneur's business model approach provides a conceptual framework to diagnose key business areas in a systemic way. The use of the cross-impact method as an in-depth diagnostic method allows the detection of significant links between variables describing different areas of the business model. In this way, we obtain two very important dimensions of connections between model variables. The first link is the relationship created at the level of individual components of the business model canvas. Variables are classified according to the canvas components and their meanings result from the links between the components. The second type of relationship resulting directly from the cross-impact analysis provides information on the importance of individual variables in the model regardless of the canvas components. The criterion for classifying variables in this case is the strength of their interaction. The analysis of variables based on the two proposed criteria allows us to describe the business model to better understand the significance of individual variables and their relations, both for the model itself and for M&A processes. Thus, it allows us to assess the consequences of changes in the structure of the business model resulting from the creation of a new business architecture.

The biggest limitation of the proposed approach is the number of variables that can be used in the cross-impact method. As our experience shows, cooperation among experts is most effective when there is a limited number of variables (up to 20). However, this is not adequate to describe the business model in a useful way for further research. However, when the number of variables exceeds 40, the effectiveness of the cooperation among experts drops significantly. It should be noted that the proposed standard list of variables is not a closed list, but only a framework proposal based on our research in the software industry. The proposed layout of variables should be treated as a starting point for discussion with industry experts for the final content of the list of variables when considering specific cases of creating a new business architecture.

From the point of view of M&A processes, it is not only the structure of key variables and their relationships that are important, but also, above all, their importance for the implementation of the strategic objectives of individual models that together form the group's business architecture. The impact of the key variables of one model on the strategic objectives of the other model is, therefore, another, third dimension of the classification of variables. As a criterion for the classification of variables, in this case, the strength of their impact on the strategic objectives of the second model is assumed. The applied approach, based on multi-criteria analysis and fuzzy logic, allows us to better describe the meaning of variables for single-level connections, at the same time reconciling very different opinions of experts involved in the consultations. This approach also works well in more complex (multi-level) acquisitions where the parent company takes over the whole company.

It has been assumed that the value (added) of the newly created business architecture will be greater the more opportunities inherent in the combination of different business models are used. The risks inherent in combinations, in turn, will be reduced by identifying and evaluating potential threats. Evaluation (quantification) of both opportunities and threats must always be carried out from the point of view of the implementation of the strategic objectives of both merged organizations, which is in accordance with Ansoff (1965) approach. Based on the perspective of the acquiring entity, we obtain a much better identification of opportunities and threats, which allows us to better estimate the potential synergistic effects of the M&A process. If we divide the strategic objectives of the models according to the recommendations of Kaplan and Norton's Balanced Scorecard concepts, with three criteria for classifying model variables, we can, in a systemic way, assess the opportunities and risks of the M&A process in terms of: market (customer), operational processes, and development.

In accordance with our approach, outlining a picture of the synergy potential in the areas of market, operational processes, and development becomes the starting point for quantifying the value (added) of a combination of business models. In the context of the effective management of synergies, the financial perspective becomes more important if the expected synergies are close to the potential synergies (reduced by integration costs) in other areas, which is in line with the relationship between several values of synergy proposed by Fiorentino and Garzella (2015). Only then can the values of key financial parameters in the M&A process be estimated with a sufficiently good approximation (e.g., the discount rate value for estimating NPV or WACC). This is important because a mismatch between cash flows and discount rates results in the overvaluation of the potential acquisitions (Mukherjee et al., 2004). The proposed approach to the study of synergy effects is, therefore, an approach combining qualitative and quantitative analyses. The results of the qualitative analysis are, in the proposed approach, a source of interpretation for the quantitative results, which is in line with the demands expressed by Perron and Gillespie (2015).

Our research proves that the combination of VAR and INT creates revenue and cost synergies in three areas (market, operating processes, and development), which adds to the statement by Capron (1999) that synergies based on income and cost cannot be related to one type of action after the acquisition.

### 5.1 Recommendations for business

The first step in applying the proposed approach to the analysis of potential M&A effects for specific organizations is to assign each company to a typical business model in the industry, and then verify the list of standard variables. This verification covers both the number of variables and their detailed descriptions (with reference to the specific case of the M&A process). The typical business models in the software industry, developed on the basis of surveys and interviews, together with the proposal of standard lists of key variables, constitute the starting point for the system procedure proposed here to assess the potential effects of combinations (or divisions) of companies. Based on typical business models in the targeted industry, it is possible to identify mutual strengths and weaknesses among any pair of models as well as potential opportunities and threats when combining companies with different business models. Thus, maximization of the added value of the M&A process can be achieved in several scenarios. The first scenario may assume searching for such a combination of business models (i.e., searching for companies classified under a specific business model), in which it will be possible to use as many opportunities as possible with acceptable threats. The second scenario, according to the min-max principle, prefers business model combinations in which the level of risk will be the lowest when the assumed level of benefits (opportunities) from the merger is reached. The proposed list of potential opportunities and threats of combining two selected models (VAR and INT models) in the context of predefined strategic objectives may constitute a reference point (roadmap) for in-depth risk analysis of building such business architectures in practice.

An equally important aspect of the approach proposed here is the structuring of decision making within the scope of M&A processes. The proposed methodology systematizes both data acquisition processes, the way tools (methods) are used, and the evaluation of the results obtained at each stage of the decision-making process. Structuring this type of decision-making process allows us to build a company knowledge base, where key information characterizing premises, context, and effects of acquisition decisions can be registered. Such knowledge, together with the appropriate mechanisms to access it, may constitute a strategic resource for the organization in building a competitive position in the market. The answer to the third research question posed earlier may, therefore, be considered in a broader context. Knowledge of the influence of different business models on the added value of a group of companies may not only be useful in decision making, but may also be a strategic resource for the company.

## Declarations

### Author contribution statement

J. Wartini-Twardowska, Z. Twardowski: Conceived and designed the experiments; Performed the experiments; Analyzed and interpreted the data; Contributed reagents, materials, analysis tools or data.

### Funding statement

This work was financially supported by University of Economics in Katowice - Minister of Science and Higher Education, Poland - Research potential.

### Competing interest statement

The authors declare no conflict of interest.

## Additional information

No additional information is available for this paper.

## Appendix A Supplementary data

Market objectives of the VAR Market objectives of the VAR

Market objectives of the INT Market objectives of the INT

Operating process of the VAR Operating process of the VAR

Operating process of the INT Operating process of the INT

Development objectives of the VAR Development objectives of the VAR

Development objectives of the INT Development objectives of the INT

Ranking Ranking
